# The SWI/SNF subunit ARID1B is important for regenerative ability of hematopoietic stem cells in normal hematopoiesis

**DOI:** 10.1371/journal.pone.0312616

**Published:** 2024-10-24

**Authors:** Olivia Arnold, Theresa Bluemn, Cary Stelloh, Sridhar Rao, Nan Zhu

**Affiliations:** 1 Versiti Blood Research Institute, Milwaukee, Wisconsin, United States of America; 2 Department of Cell Biology, Neurobiology and Anatomy, Medical College of Wisconsin, Milwaukee, Wisconsin, United States of America; 3 Department of Pediatrics, Division of Hematology/Oncology/Transplantation, Medical College of Wisconsin, Milwaukee, Wisconsin, United States of America; 4 Synthetic Biology Program, J Craig Venter Institute, La Jolla, California, United States of America; Stanford University School of Medicine, UNITED STATES OF AMERICA

## Abstract

The Switch/Sugar non-fermenting (SWI/SNF) nucleosome remodeling complexes are essential for normal hematopoiesis. The Brg1/Brm associated factor (BAF) is a form of mammalian SWI/SNF that is distinguished by the presence of either ARID1A or ARID1B protein. In this study, we used hematopoietic specific Cre mouse models to assess the function of Arid1b in blood development. We found Arid1b loss did not affect steady state hematopoiesis or hematopoietic regeneration. Nonetheless, Arid1b null hematopoietic stem and progenitor cells have reduced ability to reconstitute hematopoietic system compared to wild type cells. Overall, our data indicate Arid1b is largely dispensable for normal hematopoiesis but impairs the regenerative ability of HSPCs.

## Introduction

The Switch/Sugar Non-Fermenting (SWI/SNF) nucleosome remodeling complexes utilize the energy from ATP to disrupt nucleosome DNA interactions, resulting in altered chromatin accessibility and ultimately changes target gene transcription [1, 2]. SWI/SNF is vital in various developmental and differentiation processes [3]. They also play important roles in carcinogenesis, as mutations are found in over 25% of all cancers [4]. The Brg1/Brm associated factor (BAF) complex is one of the mammalian forms of SWI/SNF and is defined by the presence of either one of the two mutually exclusive ARID1 subunits, ARID1A or ARID1B. Previous work has shown that ARID1A is indispensable for normal hematopoiesis as its loss results in increased phenotypical hematopoietic stem and progenitor cells (HSPCs), loss of HSC quiescence, and reduction of all mature blood cell types during steady state [5], as well as defective reconstitution capabilities [5]. ARID1B has been shown to promote acute myeloid leukemia leukemogenesis [6]. In this study, we utilized the hematopoietic specific VavCre and Mx1Cre models to characterize the role of ARID1B in normal hematopoiesis. We found Arid1b is dispensable for steady state and hematopoietic regeneration. However, Arid1b loss led to impairment ability of HSPC self-renewal and differentiation in comparison to wild type in both the VavCre and Mx1Cre models.

## Materials and methods

### Animal use

Husbandry and housing of mice was provided by the Biomedical Research center at the Medical College of Wisconsin (MCW). All mouse experiments were approved by the Animal Care and Use Committee at MCW. Arid1b^fl/fl^ mice were a gift from Dr. Hao Zhu [7] (C57BL/6J background) and were bred with VavCre or Mx1Cre mice (Jackson Laboratory, Bar Harbor, ME, USA). CD45.1^+^ recipient mice were purchased from the Charles Rivers NCI (Frederick, MD, USA). PCR was used to determine the status of the floxed allele, as well as the presence of Cre. Genotyping primers were ordered from IDT (**Table 1**). Mice were genotyped by tail sample at 3 weeks of age and confirmed via peripheral blood at 6–8 weeks old prior to experimentation.

**Table 1:** Genotyping primers.

Primer	Sequence
Arid1b F	GAGAAGTGGCCACTCTGAGTA
Arid1b Flox R	AAGCATGGTTTGAAAGCCACAG
Arid1b Deleted R	GGACTCTTGTATATTTGGCAGG
Cre F	CGTATAGCCGAAATTGCCAG
Cre R	CAAAACAGGTAGTTATTCGG

### Blood collection and assessment

Peripheral blood for all experiments was collected via submental bleed every four weeks and at takedown. No anesthesia was given for submental bleed which involves momentary discomfort to the animals. Blood counts were analyzed on the SCIL ABC Animal Blood Counter (Viernheim, Germany). Red blood cells were lysed using BD Pharm Lyse Buffer (555899, BD Biosciences, Franklin Lakes, NJ, USA).

### Bone marrow transplantation

Whole bone marrow was harvested from femurs, tibias, and pelvic bones of CD45.2^+^ 8–10 week-old Arid1b^fl/fl^Cre^+^ or Cre^+^ control mice. Mice were sacrificed by CO2 asphyxiation followed by cervical dislocation. Red blood cells were lysed using BD Pharm Lyse Buffer (555899, BD Biosciences, Franklin Lakes, NJ, USA). Whole bone marrow was transplanted into lethally irradiated (950rads) CD45.1^+^ female recipient mice at 8–9 weeks of age. For non-competitive transplantations, 2 million donor cells were transplanted into recipients. For competitive transplantations, 1 million donor cells were mixed with 1 million CD45.1^+^ competitor cells and transplanted into recipients. Transplantations were conducted through tail vein (cohort 1, VavCre only) or retro-orbital injection (cohorts 2–3 in VavCre, 1–3 Mx1Cre). No anesthesia was given for tail vein injection which involves transient and minimal discomfort to the animals. Isoflurane anesthesia was given for retro-orbital injection. For Mx1Cre mice, excision was induced 4 weeks post-transplantation by intraperitoneal injection of pIpC (tlrl-pic-5, Invivogen, San Diego, CA, USA) at a dose of 12.5mg/g of body weight three times over one week. No anesthesia was given for intraperitoneal injection which causes transient and minimal discomfort to the animals.

### Flow cytometry analysis

Flow cytometry analysis was conducted on the BD LSRII or Celesta cytometers (BD Biosciences, San Jose, CA, USA). Antibodies were purchased from Biolegend, BD Bioscience, and Invitrogen (listed in **Table 2**). Staining cocktails were made in PBS ^+^ 2% FBS.

**Table 2:** Flow cytometry antibodies.

Target	Catalog Number	Company
Streptavidin	405208	Biolegend
cKit (CD117)	553356	BD Biosciences
cKit (CD117)	105813	Biolegend
Sca1 (Ly6A/E)	108114	Biolegend
Sca1 (Ly6A/E)	108123	Biolegend
CD48	103403	Biolegend
CD150	115903	Biolegend
CD34	11-0341-82	Invitrogen
FcγRII/III	101307	Biolegend
IL7Rα	135009	Biolegend
FLT3	135309	Biolegend
Mac1	101212	Biolegend
Gr1	108406	Biolegend
B220	103222	Biolegend
CD3	100206	Biolegend
CD45.1	110722	Biolegend
CD45.2	109828	Biolegend
CD45.2	109815	Biolegend
Biotin Lineage Panel	133307	Biolegend
Biotin CD45.1	110703	Biolegend

### Statistics

GraphPad Prism (version 8.2.1) was used to perform statistics. Error bars in all graphs represents mean with SD. Unpaired two-sided t-tests were used. A P value of ≤ 0.05 was considered significant.

## Results and discussion

**Fig 1:**
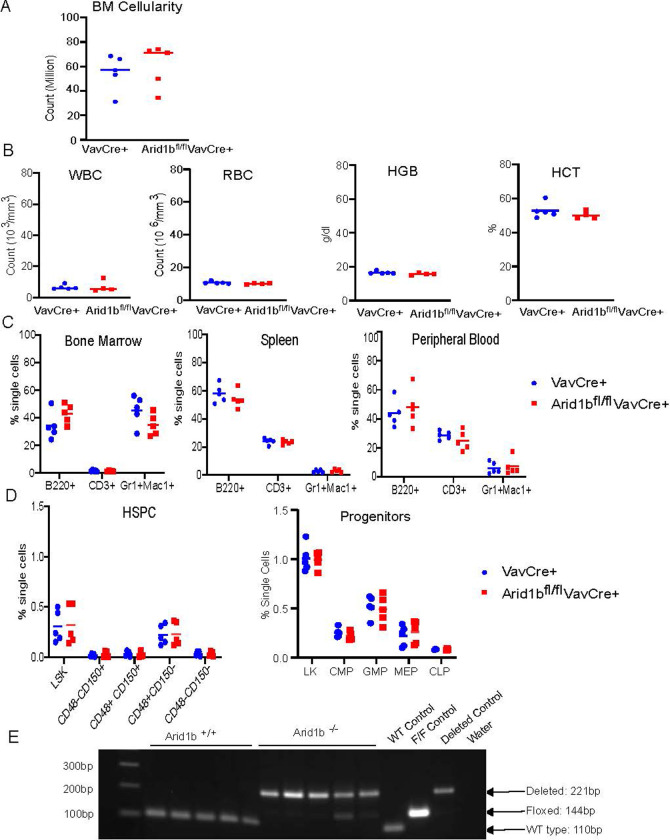
Arid1b is dispensable for steady state hematopoiesis. Effect of loss of Arid1b on steady state hematopoiesis in the VavCre model (A) Total bone marrow cellularity of both tibias and femurs at 8 weeks (B) white blood cells (WBC), red blood cells (RBC), hemoglobin (HGB), and hematocrit (HCT) in the peripheral blood. (C) Percentages of mature cell populations in the bone marrow (left), spleen (middle), peripheral blood (right). (D) Percentages of HSCs (left) and progenitors (right) in the bone marrow. (E) deletion confirmation in the bone marrow. Arid1b knockout could not be confirmed at the protein level due to reagent limitations. Data is from 5 Arid1b wild type or knockout mice. P values were determined using an unpaired 2- tailed T-Test. *P<0.05, **P<0.005, ***P<0.0005, ****P<0.00005.

We crossed a conditional Arid1b mouse model [7] (C57BL/6J to the hematopoietic specific constitutive VavCre or inducible Mx1Cre to assess the role of Arid1b in steady state hematopoiesis. Bone marrow (BM) cellularity was not different between Arid1b^fl/fl^VavCre^+^ mice and VavCre^+^ controls at 8 weeks of age (**Fig 1A**). Further, we did not observe significant defects in white blood cell, red blood cell, hemoglobin, or hematocrit (hereafter referred to as complete blood count (CBC)) in the peripheral blood (PB) in Arid1b^fl/fl^VavCre^+^ compared to VavCre^+^ mice (**Fig 1B**). Flow cytometry showed no significant differences in mature myeloid (Mac1^+^/Gr1^+^), B cells (B220^+^), or T cells (CD3^+^) in the BM, Spleen (SPL), or PB (**Fig 1C**), nor did we observe differences in hematopoietic stem cells (HSCs), Common Myeloid Progenitors (CMPs), Granulocyte/Macrophage progenitors (GMP), Megakaryocyte/Erythrocyte progenitors (MEPs), and Common Lymphoid Progenitors (CLPs) in the BM of Arid1b^fl/fl^VavCre^+^ mice compared to VavCre^+^ controls (**Fig 1D**). Deletion of Arid1b was confirmed in the BM at takedown (**Fig 1E**). Arid1b knockout could not be confirmed at the protein level due to reagent limitations.

The Mx1Cre model was used to understand the role of Arid1b in adult hematopoiesis. Deletion of Arid1b was induced at 9 weeks of age via pIpC administration. There were largely no significant differences in CBC and mature cell populations between Arid1b^fl/fl^Mx1Cre^+^ and Mx1Cre^+^ controls in the PB over time (**S3A–S3B Fig**). Additionally, we did not observe significant differences in mature cells in the BM or SPL (**S3C Fig**), nor in HSPCs in the BM of Arid1b^fl/fl^Mx1Cre^+^ mice compared to Mx1Cre^+^ controls (**S3D Fig**). Deletion was confirmed in the BM at takedown (**S3E Fig**). Taken together, data from both our VavCre and Mx1Cre models showed that Arid1b is dispensable for steady state hematopoiesis.

**Fig 2:**
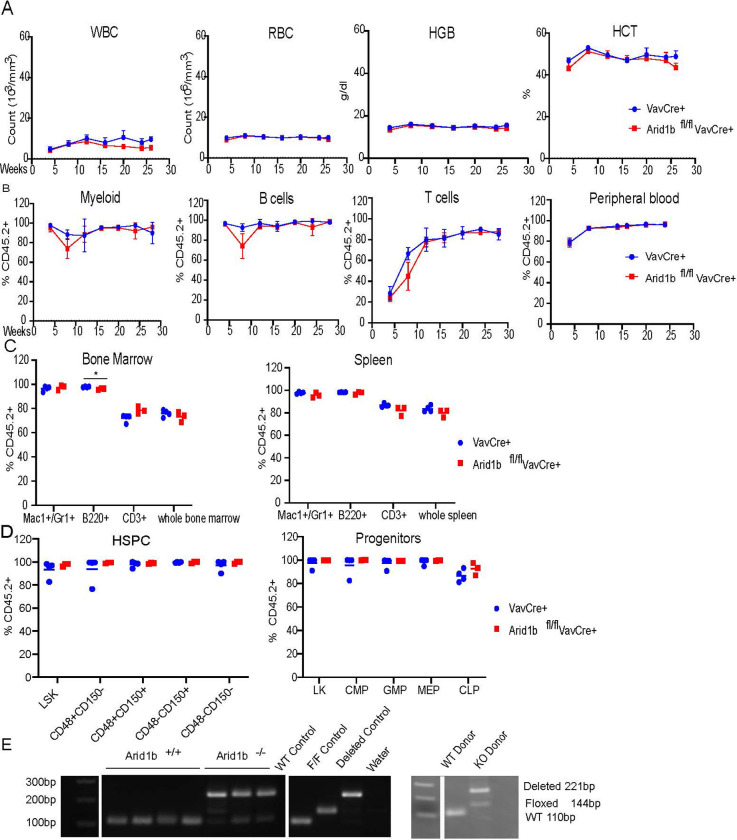
HSC reconstitution does not require Arid1b. Non-competitive transplantations in the VavCre model (A) peripheral blood complete blood count over time (B) donor chimerism of mature cells in the peripheral blood overtime (C) donor derived mature cells in the bone marrow and spleen at takedown (D) donor chimerism of HSPC populations in the bone marrow at takedown. (E) genotyping of bone marrow samples at takedown. Gel images from the same gel were sliced together as indicated by white gaps respectively for both the left and the right image. Arid1b knockout could not be confirmed at the protein level due to reagent limitations. Data shown is from one cohort, one additional cohort (n = 4 WT and 3 KO) was also assessed. P values were determined using an unpaired 2- tailed T-Test. *P<0.05, **P<0.005, ***P<0.0005, ****P<0.00005.

To understand the role of Arid1b in hematopoietic regeneration, we conducted competitive and non-competitive transplantations. In the non-competitive transplantations, we injected CD45.2^+^ Arid1b^fl/fl^Cre^+^ or Cre^+^ whole BM cells into lethally irradiated, CD45.1^+^ recipients. Deletion of Arid1b was induced 4 weeks post-transplantation in the Mx1Cre model. We did not observe significant defects in CBC or the mature blood cell populations in either Arid1b^fl/fl^VavCre^+^ nor Arid1b^fl/fl^Mx1Cre^+^ mice compared to Cre^+^ controls (**Figs 2A–2B and S4A–S4B**). Upon takedown, there were no significant differences in total donor cells nor in donor chimerism of mature myeloid, B cells, or T cells in the BM and SPL of the Arid1b deleted versus Cre^+^ controls (**Figs 2C and S4C**), nor were there changes in the CD45.2^+^ HSPCs in the BM of Arid1b^fl/fl^VavCre^+^ mice compared to controls (**Fig 2D**). We did observe marginal, although significant, differences in the BM CD45.2^+^ HSCs of Arid1b^fl/fl^Mx1Cre^+^ mice (padj< 0.05), but not in the donor progenitors at takedown (**S4D Fig**). Knockout was confirmed in the BM at takedown for both models (**Figs 2E and S4E**). Arid1b knockout could not be confirmed at the protein level due to reagent limitations. Additionally, we did not observe any significant changes in BM cellularity or in the absolute number of CD45.2+ mature or HSPCs between Arid1b knockout versus controls in VavCre or MxCre non-competitive transplantations (**S5 Fig**). Collectively, this data shows that Arid1b null HSCs can fully reconstitute the BM in the non-competitive transplantation setting.

**Fig 3:**
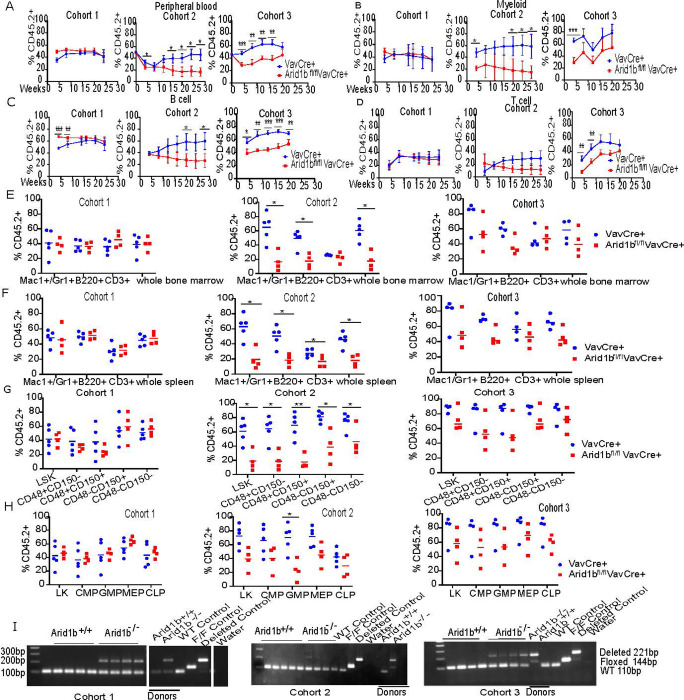
Arid1b loss impairs HSC reconstitution in competitive transplantations. (A) Total CD45.2^+^ donor cells in the PB over time (B-D) donor derived mature myeloid (B), B cells (C) and T cells (D) in the PB over time. (E-F) donor derived mature cells in the BM (E) and SPL (F) at takedown (20–26 weeks). (G-H) donor derived HSPCs in the BM at takedown. (I) genotyping of BM at takedown. Left panel: gel images from the same gel were sliced together as indicated by white gaps. Arid1b knockout could not be confirmed at the protein level due to reagent limitations. Data is from three separate cohorts of n = 4–5 mice each for knockout or wild type conditions. Each cohort was transplanted with whole bone marrow from one wild type or one knockout donor. Sex of the donor mice used: cohort 1 VavCre control male and Arid1b^f/f^VavCre female, cohort 2 VavCre control female and Arid1b^f/f^VavCre male, and cohort 3 VavCre control male and Arid1b^f/f^VavCre female. P values were determined using an unpaired 2- tailed T-Test. *P<0.05, **P<0.005, ***P<0.0005, ****P<0.00005.

We next assessed the regenerative ability of Arid1b null HSPCs using competitive transplantation. To do this, whole BM from CD45.2^+^ donor (Arid1b^fl/fl^Cre^+^ or Cre^+^ control) and CD45.1^+^ competitor mice was mixed 50:50 and transplanted into lethally irradiated recipients. In our VavCre model, we observed significantly lower donor chimerism in the PB (**Figs 3A and S6A**) of Arid1b null versus control mice indicating decreased regenerative potential. We also observed significant decreases in the chimerism of mature myeloid and B cells, but not T cells in the PB of Arid1b^fl/fl^VavCre^+^ mice versus VavCre^+^ controls (**Figs 3B–3D and S6A**). At takedown, we found significant defects in donor myeloid and B cells in the BM and SPL, as well as T cells in the SPL, in Arid1b knockout mice (**Figs 3E, 3F and S6B**). There were also significant decreases in donor HSCs and GMPs, with trends towards decreases in the CMPs, MEPs, and CLPs in the BM of knockout mice (**Figs 3G, 3H and S6C**). Deletion was confirmed in the BM (**Fig 3I**). Arid1b knockout could not be confirmed at the protein level due to reagent limitations.

**Fig 4:**
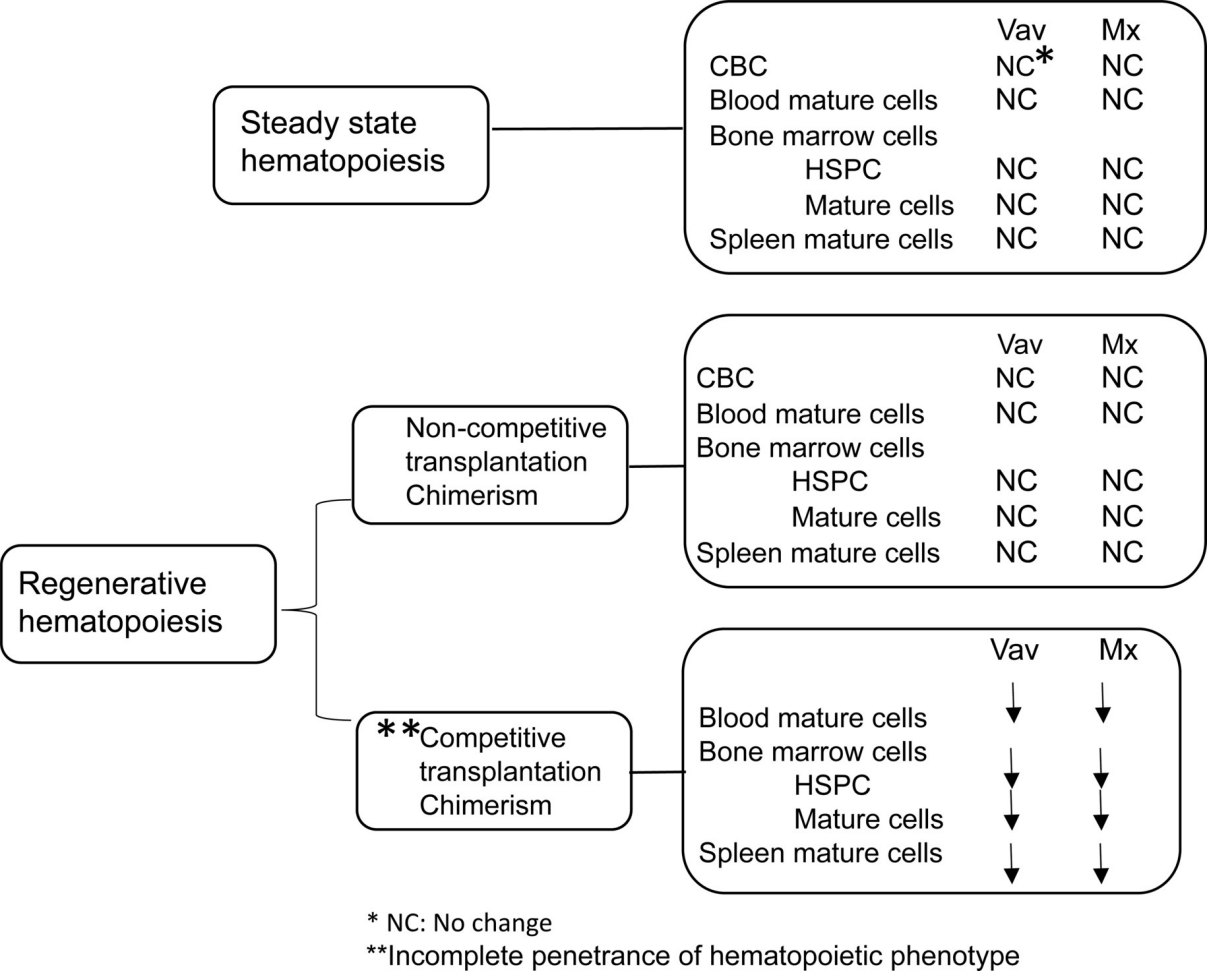
Summary of Arid1b loss phenotypes in normal hematopoiesis. Diagram showing summarized results of effect of Arid1b loss on steady state and regenerative hematopoiesis in Vav and Mx Cre models.

We observed a similar phenotype in our Mx1Cre transplantations (**S7A–S7I Fig**), in which we found decreased chimerism overall and of mature myeloid cells in the PB overtime (**S7B Fig**), as well as in takedown BM and SPL (**S7E and S7F Fig**) in Arid1b knockout recipient mice. We observed a trend in decreased chimerism of B cells in the PB and BM (**S7C and S7E Fig**) although statistically insignificant. Further, there were significant defects in donor HSCs, CMPs, GMPs, and MEPs in the BM of Arid1b^fl/fl^Mx1Cre^+^ mice compared to Mx1Cre^+^ controls (**S7G–S7H Fig**). Deletion was confirmed at takedown (**S7I Fig**). Interestingly, the effects of Arid1b knockout seemed to occur with incomplete penetrance in both models, as the observed phenotypes occurred in the majority, but not all, of our transplanted mice, dependent upon the donor mouse used. Together, these data (summarized in Fig 4) suggest that Arid1b loss impairs the regenerative ability of HSPCs against wild type cells, leading to decreased self-renewal and differentiation in competitive transplantations with incomplete penetrance.

While this study was ongoing, another group published a characterization of Arid1b loss using the Mx1Cre model [8]. Their findings were largely consistent with ours, however they did not report changes in HSPCs chimerism in competitive transplantations of Arid1b knockout versus control mice. In both the VavCre and Mx1Cre models, we observed significant decreases in chimerism of HSCs and myeloid progenitors in Arid1b knockout mice compared to controls. There were additional defects in the reconstitution of CLPs and mature B cells in Arid1b^fl/fl^VavCre^+^ mice which were not observed in the Mx1Cre model. Additionally, our study revealed the role of Arid1b in competitive hematopoiesis may occur with incomplete penetrance, however the study by Madan et al [8] did not. This discrepancy may be due to the strain specific differences of the mouse models used, number of recipients assessed or number of donor mice used compared to our study. Their competitive transplantations were conducted with 2–5 recipient mice and an unknown number of donors, while we used 4–5 recipient mice in three independent cohorts with three separate donors for both the VavCre and MxCre models. The mice used on our study were on the C57BL/6J background, while Madan et al utilized the C57BL/6N sub-strain. In competitive transplantations of LSKs from C57BL/6J or C57BL/6N mice, C57BL/6N mice showed enhanced short-term repopulation of recipient blood compared to C57BL/6J mice [9]. It is possible they did not observe variations in their cohorts as we did due to this enhancement of LSK abilities inherent to C57/6N mice.

ARID1B shares high homology with ARID1A. While ARID1A loss is detrimental to hematopoiesis [5], ARIDB loss largely leaves hematopoietic development intact. Madan et al. showed that double knockout of Arid1a/b results in total bone marrow failure [8], a phenotype that is more severe than, and does not phenocopy, knockout of either subunit alone. These data suggests that ARID1A plays a more prominent role in hematopoiesis, while only partially compensating for each other’s loss. This is supported by the finding that, at the molecular level, upon the loss of ARID1A or ARID1B, overlapping and unique alterations of chromatin accessibility of genes were identified.

Overall, our study has found ARID1B is largely dispensable for normal hematopoiesis. However, its loss reduces HSPC fitness in hematopoietic regeneration. Further work is needed to understand the molecular mechanism underlying the reduced HSPC fitness upon Arid1b loss.

## Supporting information

S1 Fig Gene expression levels of three of the unique subunits of SWI/SNF BAF complex Arid1a, Arid1b and Dfp2 in hematopoiesis. Plots generated and downloaded from Haemosphere (Choi et al. Nucleic Acid Res. 2018) using data from Haemopedia RNA-seq (Choi et al. Nucleic Acid Res. 2019).
(PDF)

S2 Fig Representative flow cytometry gating schemes. Flow cytometry gating schemes for HSPC (A), progenitors (B: CMP, GMP, MEP), CLP (C) and mature cells (D).
(EPS)

S3 Fig Arid1b is dispensable for hematopoietic maintenance in Mx1Cre model. Effect of loss of Arid1b on steady state hematopoiesis in the Mx1Cre model (A) white blood cells (WBC), red blood cells (RBC), hemoglobin (HGB), and hematocrit (HCT) in the peripheral blood. (B) Percentages of mature myeloid (left), B (middle), and T (right) cell populations in the peripheral blood. (C) Donor chimerism of mature cells in the BM (left) and SPL (right) (D) HSCs (left) and progenitors (right) in the bone marrow. (E) deletion confirmation in the bone marrow. Data is from six Arid1b wild type or knockout mice. P values were determined using an unpaired 2- tailed T-Test. *P<0.05, **P<0.005, ***P<0.0005, ****P<0.00005.
(EPS)

S4 Fig Arid1b is dispensable for hematopoiesis in non-competitive transplantation in Mx1Cre model. (A) Complete blood count over time (B) donor chimerism of mature cells in the peripheral blood over time (C) donor derived mature cells in the bone marrow and spleen at takedown (D) donor derived HSPC populations in the bone marrow at takedown. (E) genotyping of bone marrow samples at takedown. Gel images from the same gel were sliced together as indicated by white gaps. Data shown is from one cohort (n = 3WT and 4KO), one additional cohort was also assessed. P values were determined using an unpaired 2- tailed T-Test. *P<0.05, **P<0.005, ***P<0.0005, ****P<0.00005.
(EPS)

S5 Fig Cellularity in non-competitive transplantations. Cellularity of bone marrow (A), absolute numbers of mature bone marrow populations (B), spleen cell populations (C), HSPC (D) and progenitors (E) in Vav and Mx non-competitive transplantations shown in Figs 2 and S4. P values were determined using an unpaired 2- tailed T-Test. *P<0.05, **P<0.005, ***P<0.0005, ****P<0.00005.
(EPS)

S6 Fig VavCre competitive transplantation combined data. (A) donor derived CD45.2+ mature myeloid, B cells and T cells in the PB over time. (B) donor derived mature cells in the BM and SPL at takedown (20–26 weeks). (C) donor derived HSPCs in the BM at takedown. Shown are combined data from three separate cohorts of n = 4–5 mice each for knockout or wild type conditions. Each cohort was transplanted with whole bone marrow from one wild type or one knockout donor. P values were determined using an unpaired 2- tailed T-Test. *P<0.05, **P<0.005, ***P<0.0005, ****P<0.00005.
(EPS)

S7 Fig Inducible deletion of Arid1b impairs HSPC function and myeloid differentiation in Mx1Cre model. (A) total CD45.2+ donor cells in the PB over time. Donor derived mature myeloid, B cells, and T cells in the PB (B-D), BM (E), and SPL (F). (G-H) donor derived BM HSPCs at takedown. (I) takedown genotyping of bone marrow. Left panel: gel images from the same gel were sliced together as indicated by white gaps. Data is from three separate cohorts of n = 2–5 mice each for knockout or wild type conditions. Sex of the donor mice used: cohort 1 MxCre control female and Arid1b^f/f^MxCre knockout female, cohort 2 MxCre control male and Arid1b^f/f^MxCre female, and cohort 3 MxCre control female and Arid1b^f/f^MxCre male. P values were determined using an unpaired 2- tailed T-Test. *P<0.05, **P<0.005, ***P<0.0005, ****P<0.00005.
(EPS)

S1 Raw image Raw images of genotyping PCRs. (PDF)

S1 Dataset (XLSX)
