# Supplementary figures and images for: The SWI/SNF subunit ARID1B is important for regenerative ability of hematopoietic stem cells in normal hematopoiesis
Source: PLoS One. 2024 Oct 24;19(10):e0312616. doi: 10.1371/journal.pone.0312616 (PMC11500929; doi:10.1371/journal.pone.0312616)

Arid1a

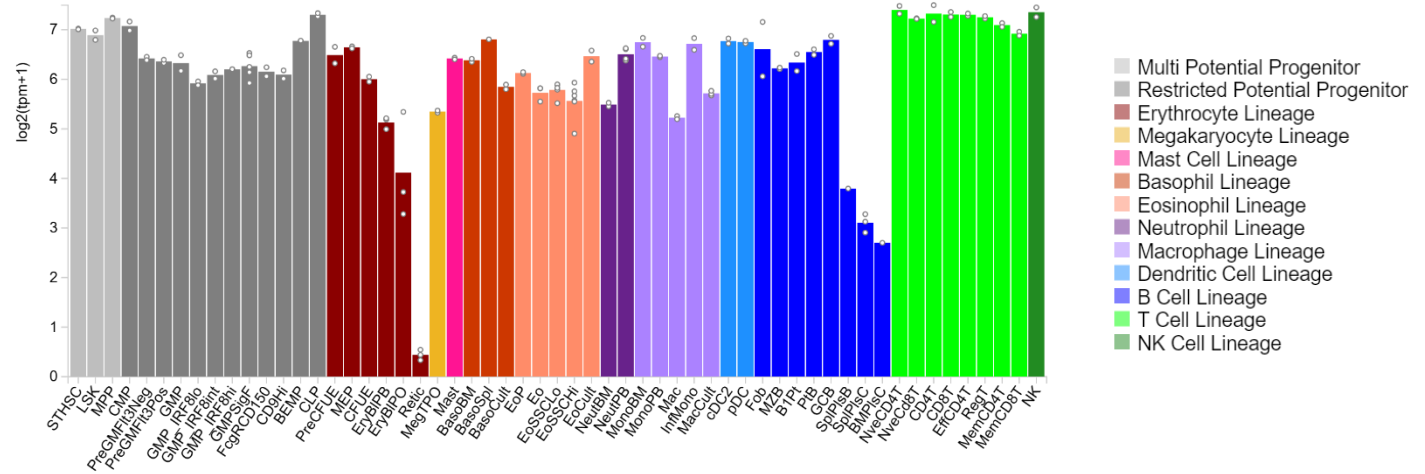

## Arid1b

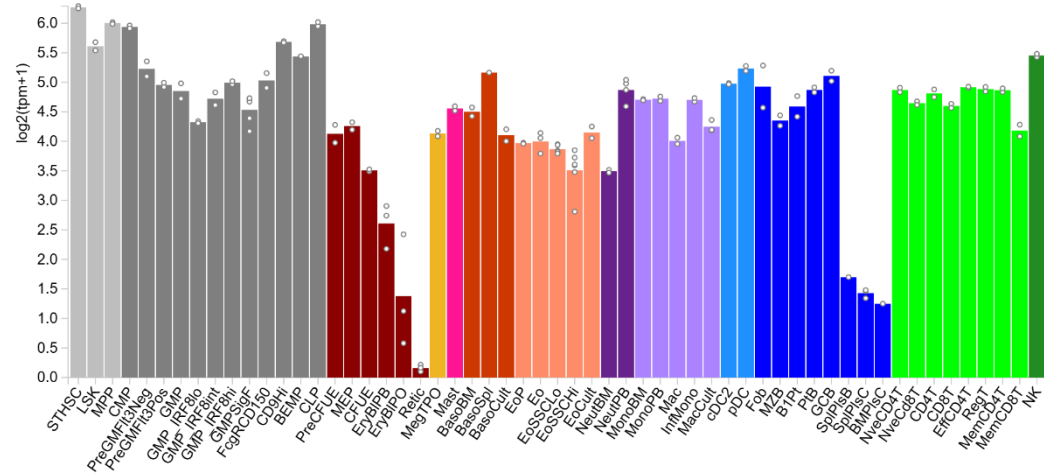

Dpf2

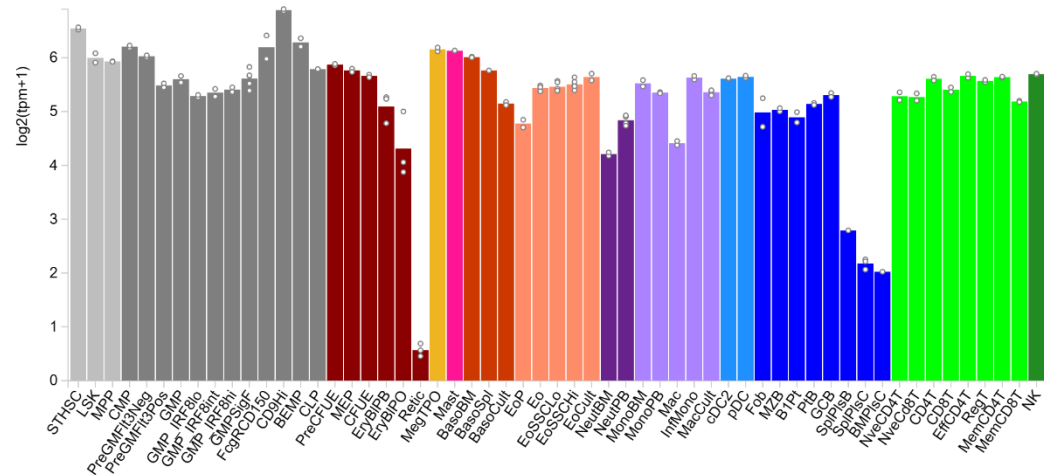

Supplement: S1 Fig — Plots generated and downloaded from Haemosphere (Choi et al. Nucleic Acid Res. 2018) using data from Haemopedia RNA-seq (Choi et al. Nucleic Acid Res. 2019). (PDF) [file pone.0312616.s001.pdf]

Fig 1E

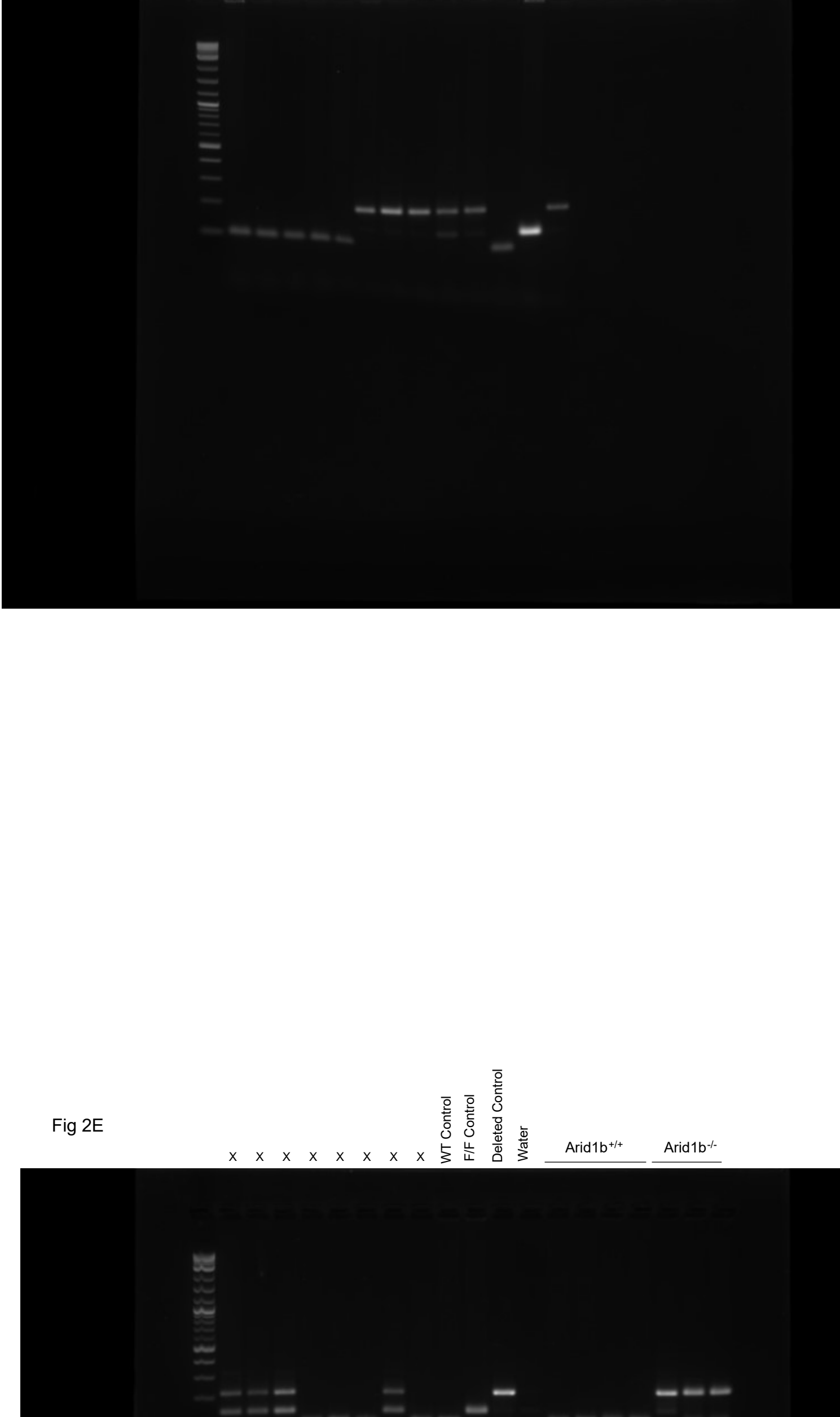

Fig 2E

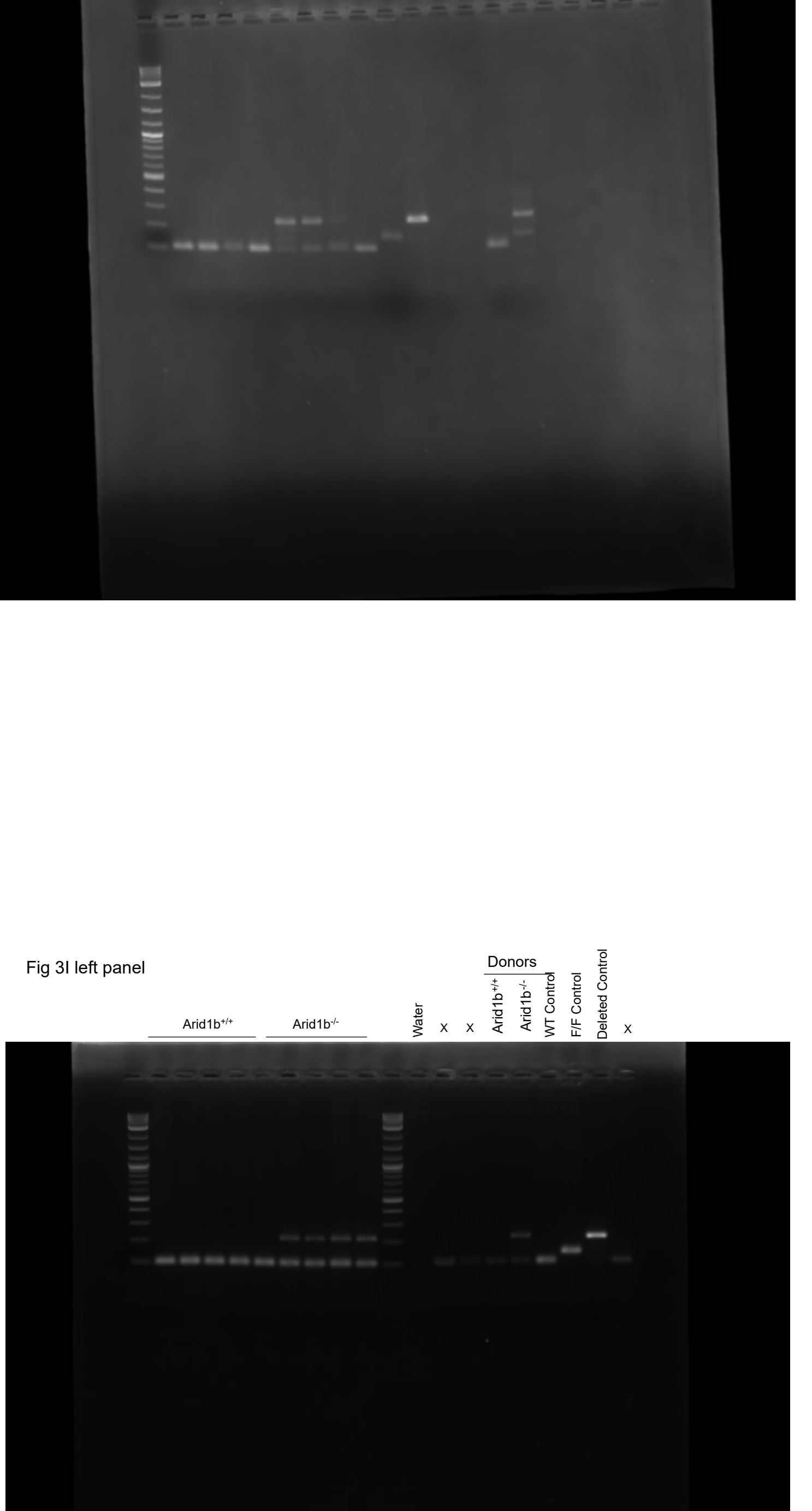

Fig 2E

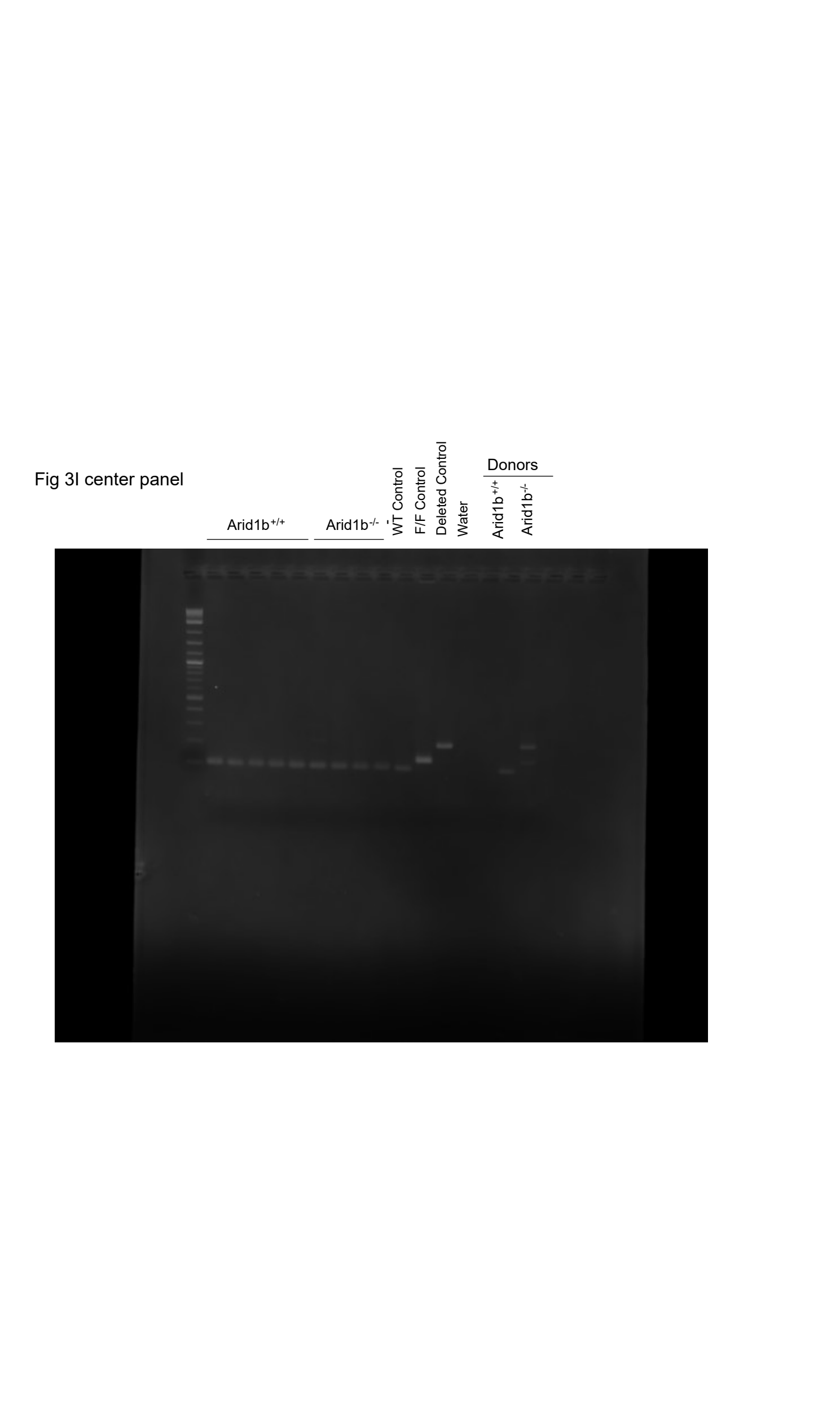

Fig 3I left panel

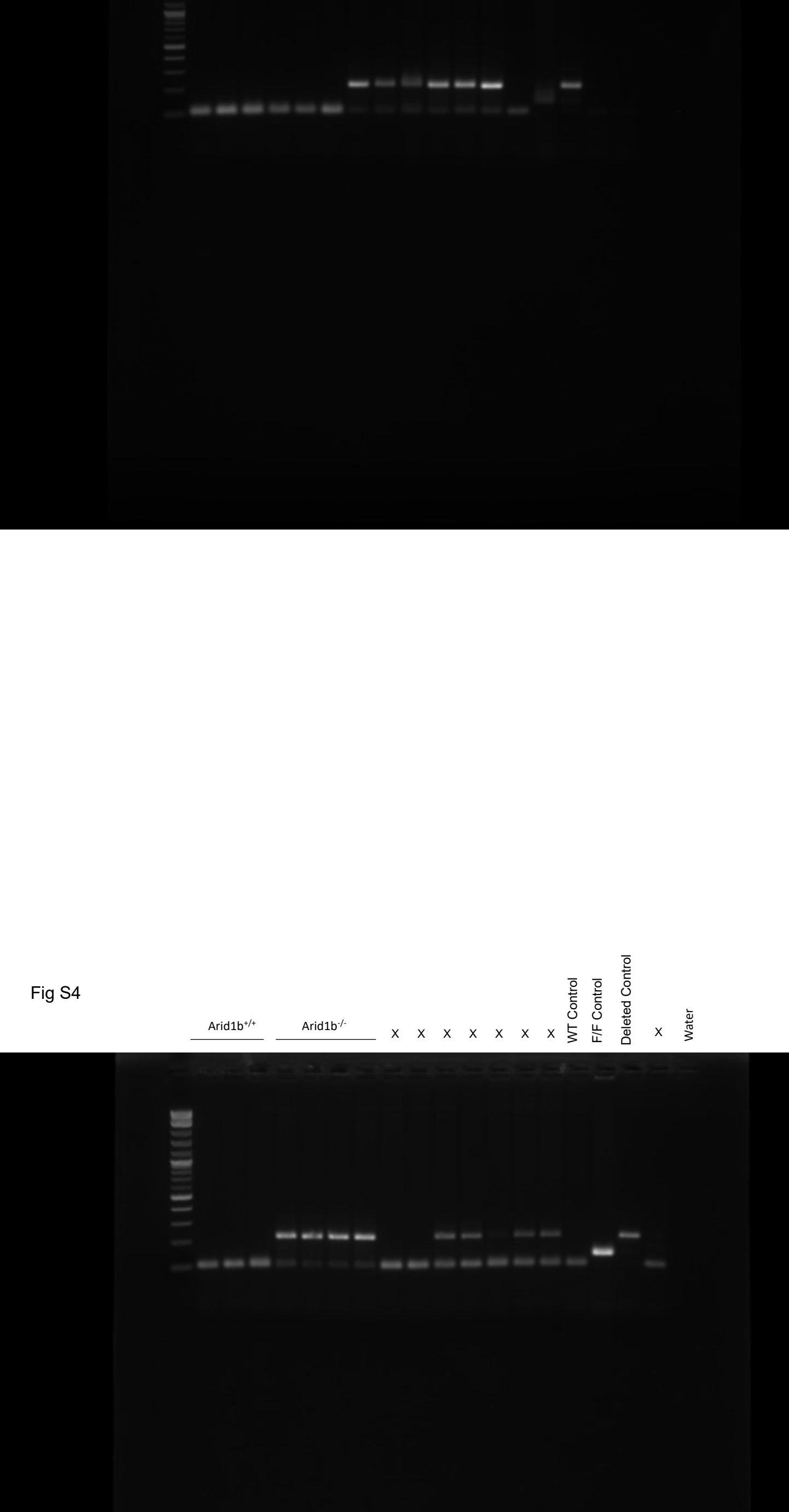

Fig S3

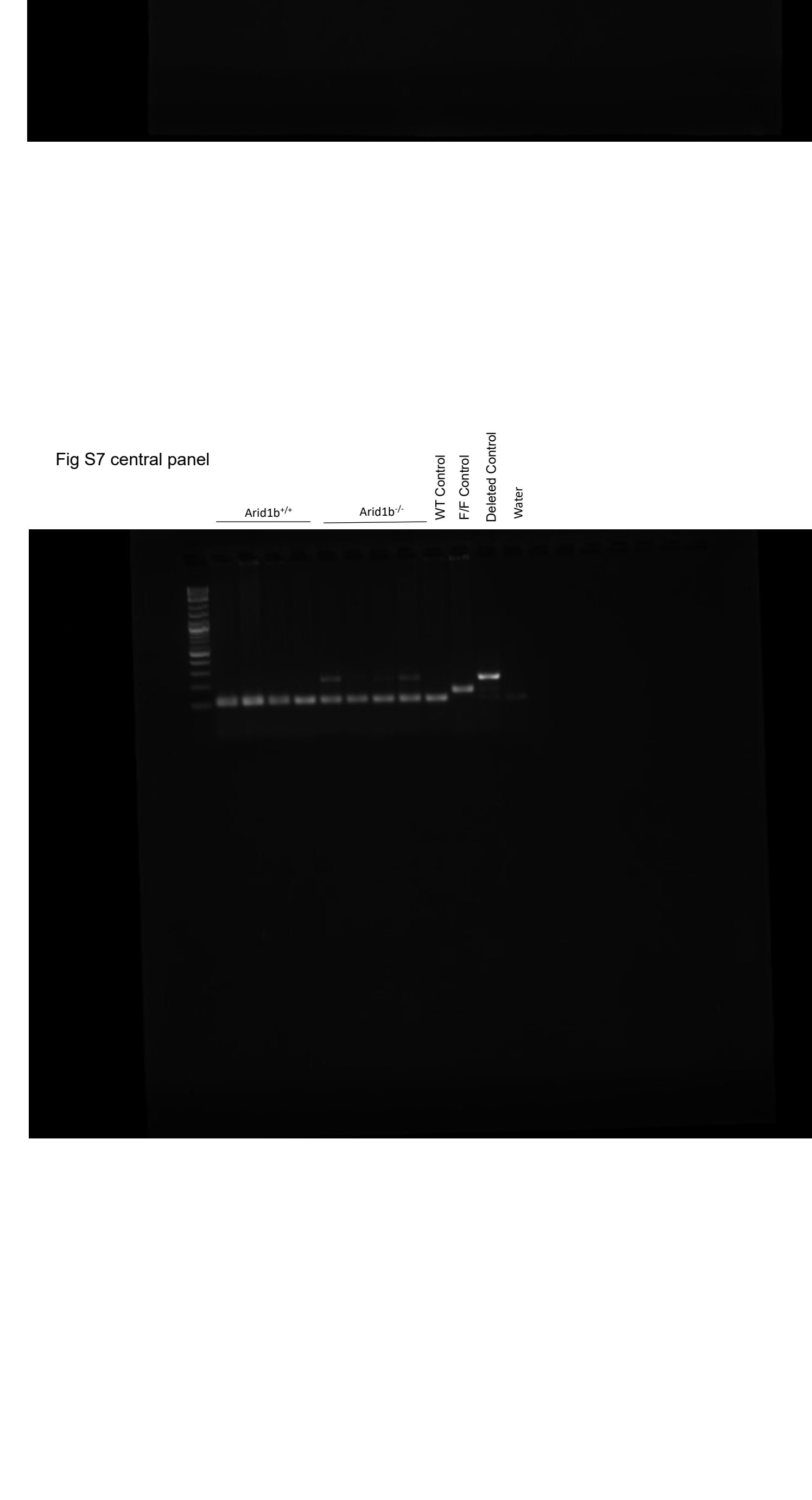

Fig S4

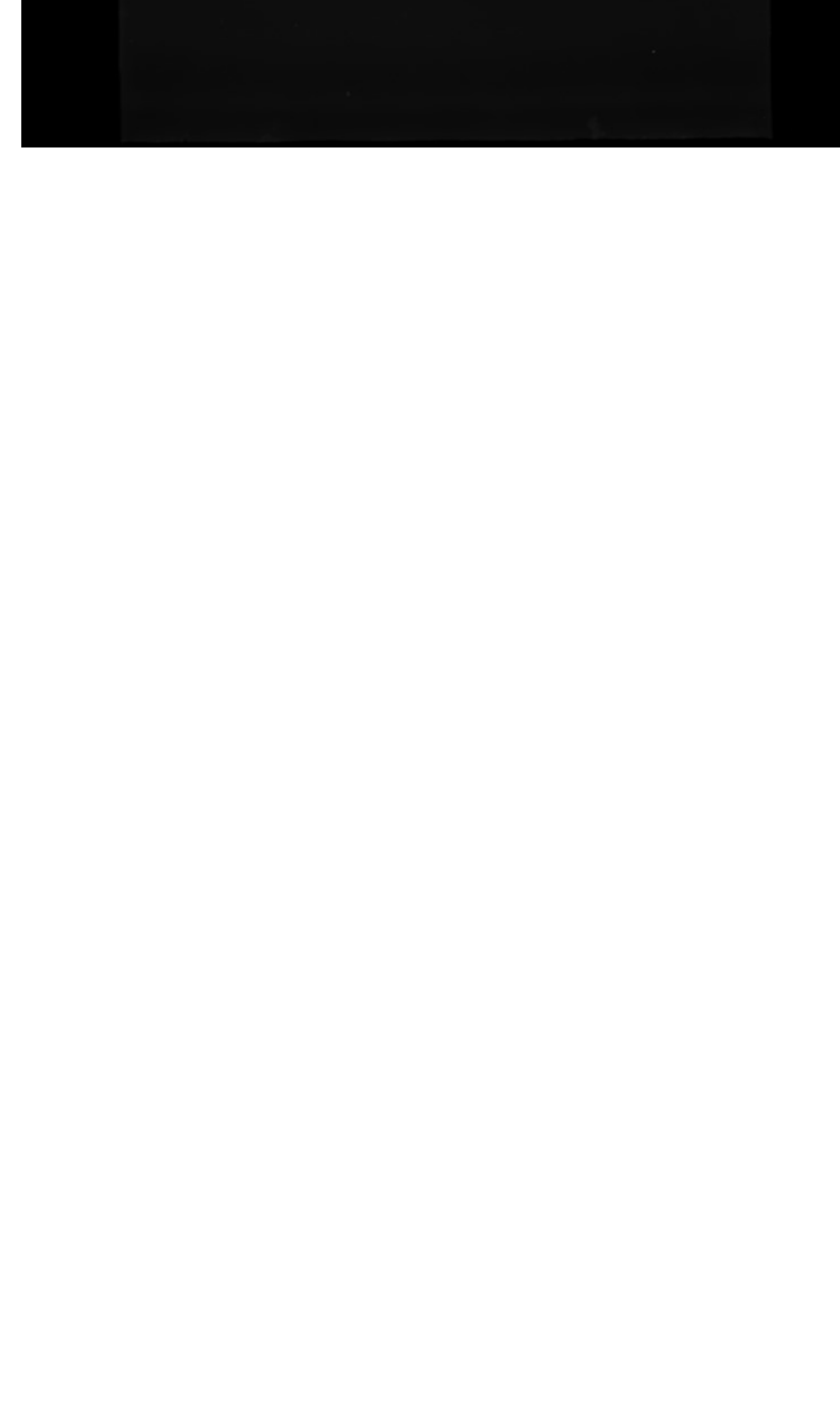

Fig S7 left panel

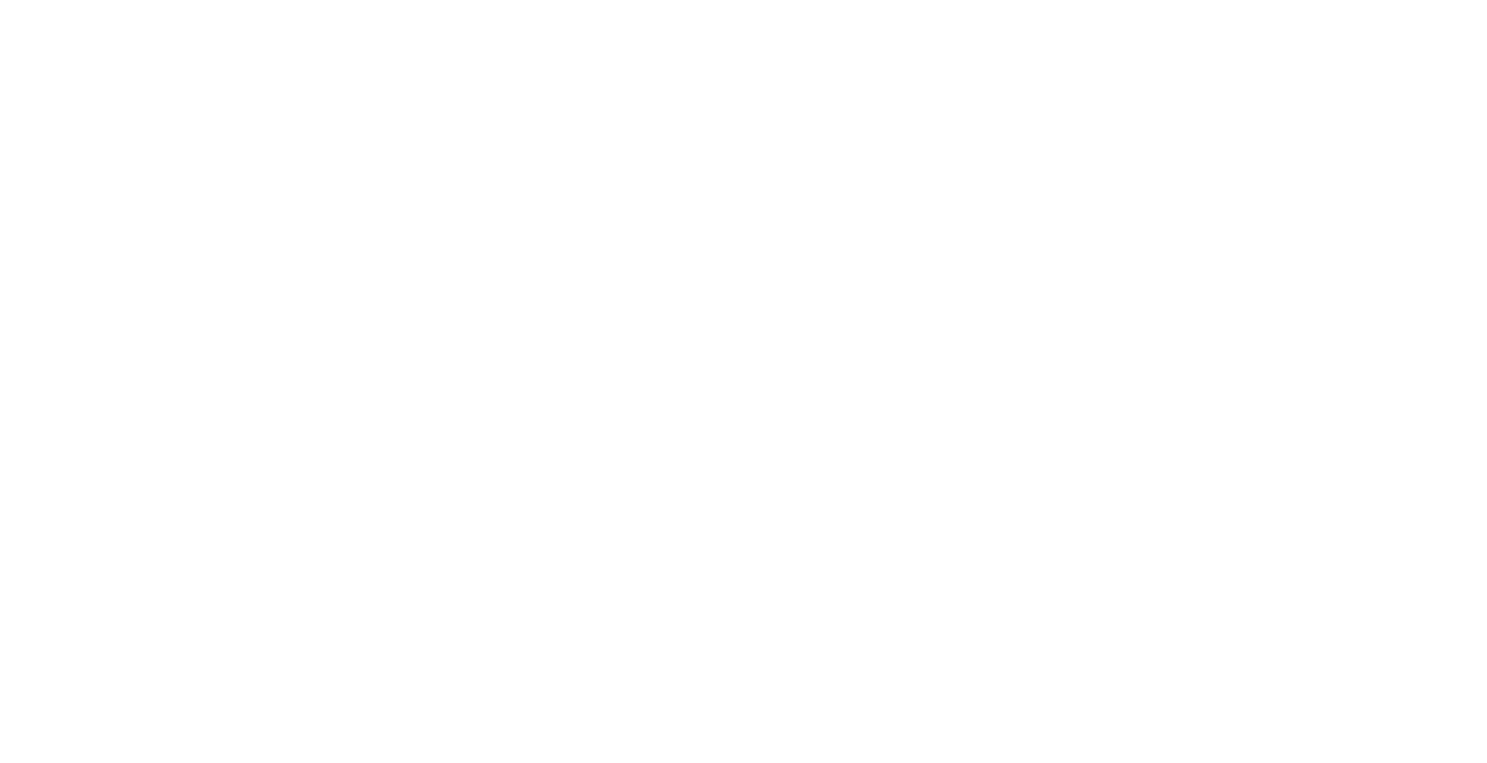

Fig S7 central panel

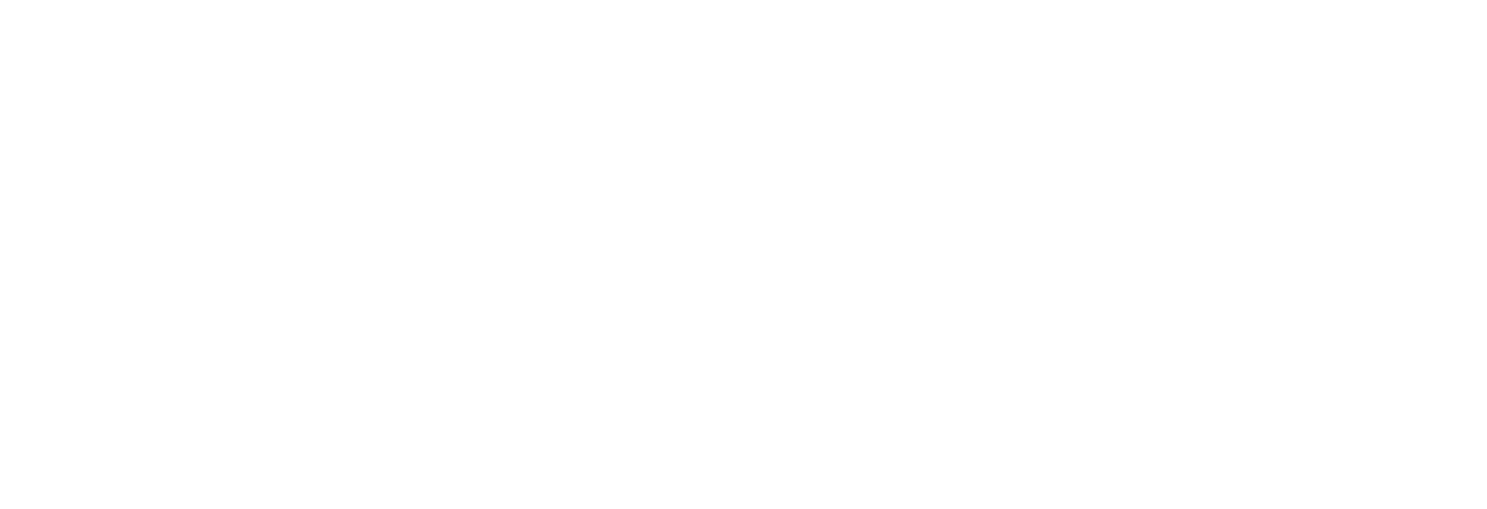

Fig S7 right panel

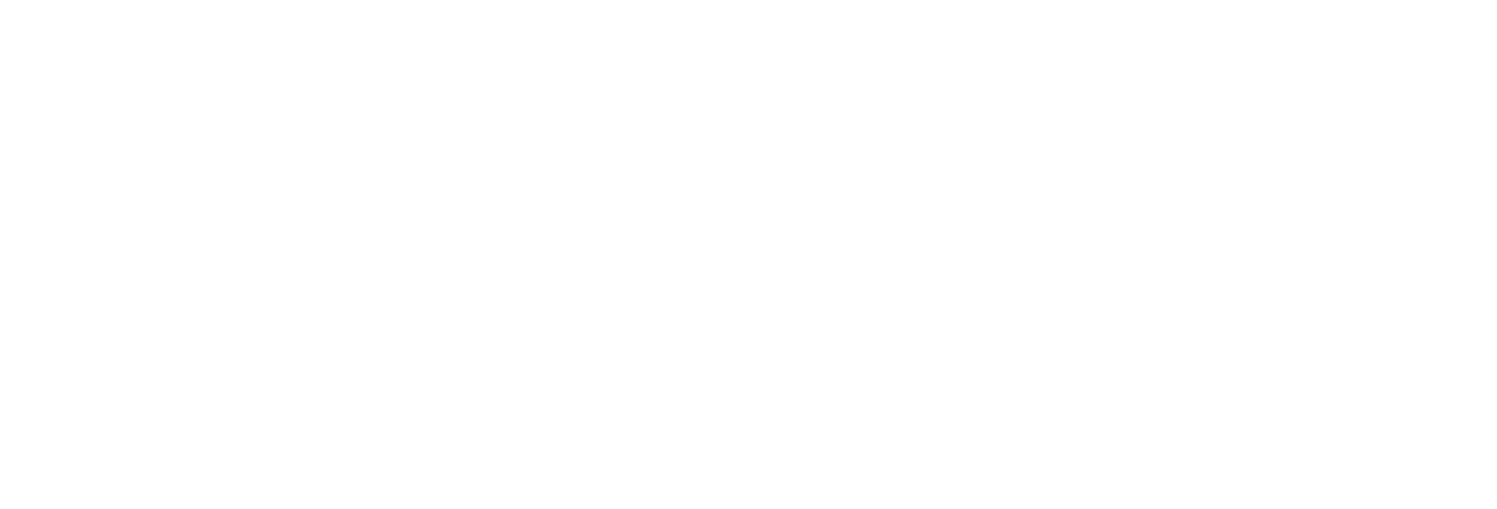

Supplement: S1 Raw image — (PDF) [file pone.0312616.s008.pdf]
